# Transcranial direct-current stimulation (tDCS) in the primary motor cortex and its effects on sensorimotor function: a quasi-experimental single-blind sham-controlled trial

**DOI:** 10.1038/s41598-021-85989-7

**Published:** 2021-03-22

**Authors:** Sergio Lerma-Lara, Marina De Cherade Montbron, Mathias Guérin, Ferran Cuenca-Martínez, Roy La Touche

**Affiliations:** 1 Departamento de Fisioterapia, Centro Superior de Estudios Universitarios La Salle, Universidad Autónoma de Madrid, C/ La Salle, No. 10, 28023 Madrid, Spain; 2 Motion in Brains Research Group, Institute of Neuroscience and Sciences of the Movement (INCIMOV), Centro Superior de Estudios Universitarios La Salle, Universidad Autónoma de Madrid, Madrid, Spain; 3 Hospital Infantil Universitario Niño Jesús, Madrid, Spain; 4 Instituto de Neurociencia y Dolor Craneofacial (INDCRAN), Madrid, Spain

**Keywords:** Neuroscience, Physiology

## Abstract

The main aim was to assess the short-term effects of active-tDCS (a-tDCS) in the primary motor cortex (anodal stimulation-M1) on sensorimotor variables. These variables included discriminative sensation through the two-point discrimination (2-PD) test, tactile acuity threshold and pressure pain threshold (PPT), and electromyographic (EMG) activity compared with a sham-tDCS (s-tDCS) in healthy individuals. A total of 100 participants were included. Fifty of the participants received the a-tDCS application of 2 mA for 20 min, whereas the remaining fifty received the s-tDCS. The 2-PD and tactile acuity threshold in thenar eminence of the hand and in the dorsal part of the foot and also, PPT and EMG activity during maximal voluntary contraction in the biceps brachii and rectus femoris were assessed before and after the tDCS application. The a-tDCS intervention was not significantly superior to the s-tDCS in any variable. However, significant within-group pre- and post-intervention differences were found in the a-tDCS, such as the tactile acuity threshold in thenar eminence of the hand, with a small effect size (*p* = .012, *d* = 0.20) and in the PPT of the rectus femoris, also with a small effect size (*p* = .001, *d* =  − 0.17). Regarding EMG activity, a trend towards greater activity was observed in participants with a-tDCS compared with s-tDCS, which showed a trend towards decreased EMG activity. In fact, although no differences were found between the groups, within-group differences were statistically significant in the biceps brachii pre- and postintervention (*p* = .023, *d* =  − 0.16, and *p* = .002, *d* = 0.18, respectively), and also in the rectus femoris, only in the a-tDCS, with a small effect size (*p* = .011, *d* =  − 0.14). This study showed no significant between-group differences in sensorimotor outcomes. A single session of tDCS in isolation appears to produce immediate effects in healthy participants on sensorimotor function; however, these effects were very small.

## Introduction

Transcranial direct-current stimulation (tDCS) devices have emerged in recent years, providing important benefits in pain management and muscle training that could be used in routine clinical practice^1^. Moreover, in the past few years, several studies have demonstrated the safety of this technique^2,3^. Bikson et al.^3^ had found no risk of brain injury within the following parameters: < 40 min; < 4 milliamperes (mA); and < 7.2 Coulombs. The safety assessment of tDCS has contributed to encouraging further studies.

Currently, the most investigated tDCS effect is cortical excitability modulation. Indeed, tDCS has been shown to be an interesting tool for increasing and also decreasing corticomotor excitability in healthy individuals and in patients with stroke when applied to the primary motor cortex (M1)^4^.

In addition, most published articles on changes in pain after electric current application on the primary motor cortex in healthy individuals focus on pain tolerance^5–7^. Almost all these studies involved inducing pain by temperature adjustment. Mariano et al.^7^, for example, had described improvements in pain tolerance with pressure and cold. Angius et al.^8^ had also observed reduced pain perception during a cold pressure test. However, they did not report any decrease in pain during exercise. Thermoalgesic information and tactile sensitivity travel along distinct afferent pathways to make afference to the central nervous system. The skin contains mechanical pressure receptors such as Ruffini's corpuscles or Merkel's discs, as well as free nerve endings that are activated after exceeding a certain threshold of activation. Both thermoalgesic and tactile information converge in that they are exteroceptive sensitivity. Epicritic tactile sensitivity travels through the dorsal column–medial lemniscus pathway while protopathic tactile sensitivity travels through the anterior spinothalamic cord. Finally, thermoalgesic sensitivity travels through the lateral spinothalamic cord^9^. In the study of Cobos et al.^10^ they consider that the modalities of mechanical pain and thermal pain may have different mechanisms due to the different times of onset after neural lesion. This seems to be in agreement with the findings found in the literature due to the difference in effects found depending on the type of pain studied. However, the effects of tDCS on pain modulation are not yet fully understood^11^.

Given very little has been assessed regarding the effects of tDCS on the pressure pain threshold (PPT) in healthy participants, PPT is an interesting focus of study to enhance our understanding of tDCS effects^12,13^. tDCS application to the M1 and any resulting changes in sensitivity have not yet been investigated and could provide interesting results.

In addition, tDCS devices have been widely used in recent years in sports-related studies. In the current literature, data have been found both for and against the effect of tDCS on strength. For example, Vargas et al.^14^ had described an increase in quadriceps strength during maximal voluntary contractions. However, Giboin et al.^15^ had shown the opposite effect from tDCS. In addition, Hendy & Kidgell^16^ performed transcranial anodal direct current anodal stimulation in asymptomatic subjects combined with standard strength training with the goal of increasing voluntary dynamic strength in wrist extensors. They found that adding anodal stimulation did not provide any significant improvement in strength enhancement compared to strength training in isolation. However, also Hendy & Kidgell^17^ found that applying transcranial anodal direct current stimulation during unilateral strength training is able to elicit strength and corticospinal excitability even in the untrained contralateral limb muscle. These contradictions in the literature highlight the need to increase our understanding of the connections between muscle activity and tDCS intervention.

Although Chhatbar et al.^18^ showed that electrical current flows through the human brain in vivo with this technique, further investigation is needed to establish and define more precisely the optimal parameters and effects of tDCS stimulation in healthy subjects and in cases of pain and muscle activity. However, the literature already presents clearer parameters for some results. Lefaucheur et al.^19^ recently developed evidence-based guidelines on the therapeutic use of tDCS which set out some parameters. For example, in neuropathic pain due to spinal cord injury, placing the anode at M1 and the cathode at the supraorbital region, 5 sessions at 2 mA for 20 min have been shown to have an effect on pain modulation. Also, placing the anode and cathode in the same area, at 1 mA, 10 sessions of 20 min have been shown to have an impact on pain reduction in fibromyalgia. However, therapeutic relevance of tDCS needs to be further explored.

The hypothesis is that the application of a-tDCS will cause greater effects in sensorimotor function (higher PPT, greater electromyographic activity and better discriminative sensation) compared to the application of s-tDCS. Due to the lack of literature on the effects of tDCS on sensory-discriminative variables as well as contradictory findings regarding the effects on pain modulation and motor variables, the main aim of the present study was to assess the short-term effects of active-tDCS (a-tDCS) on the M1 regarding sensorimotor variables such as discriminative sensation, PPT and electromyographic activity compared with a sham-tDCS (s-tDCS) in healthy individuals.

## Methods

### Study design

**Figure 1:**
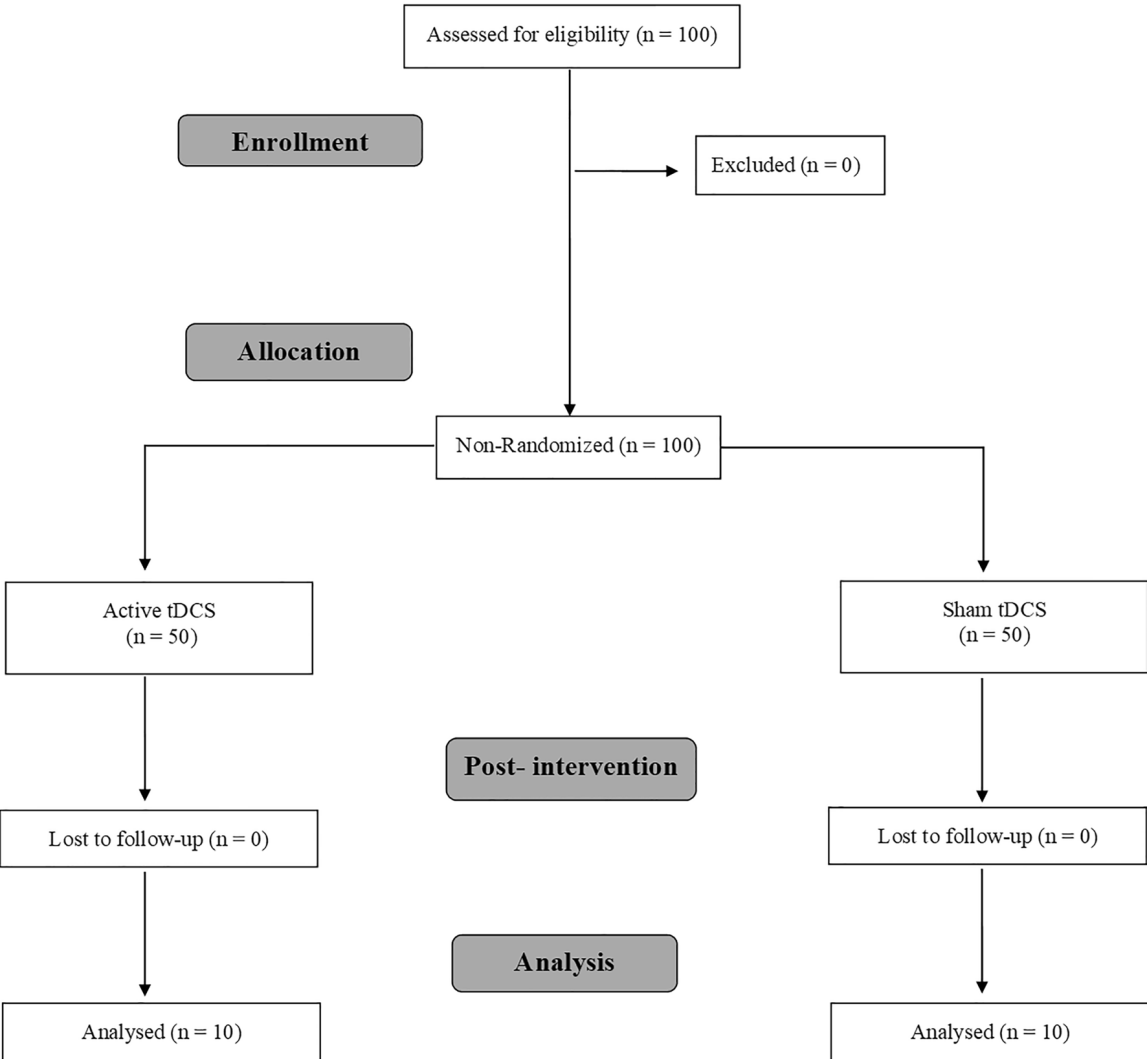
Study flow chart.

The present study was a quasi-experimental, single-blind, sham-controlled trial. A non-probabilistic sample was used. The participants were assigned to a group depending on their date of registration (Fig. 1). The first 50 were placed in the a-tDCS group and the following 50 were assigned to the s-tDCS group. The study protocol follows the Consolidated Standards of Reporting Trials statement on randomised trials of nonpharmacological treatments^20^.

All procedures were approved by the Human Research Ethics Committee of the Hospital Infantil Universitario Niño Jesús of Madrid (CI: R-0022/18). This study was registered in the United States Randomized Trials Register on clinicaltrial.gov (trial registry number: NCT04148547 (01/11/2019 Last update).

### Participant recruitment

A total of 100 asymptomatic volunteers were recruited from September 2018 to June 2019 from the local community through social media and e-mail. The inclusion criteria were as follows: (a) asymptomatic participants; and (b) men and women aged 18 to 65 years. The exclusion criteria were the following: (a) insomnia; (b) nausea; (c) headache; (d) pregnancy; (e) use of painkillers in the last 24 h; (f) presence of metal inside the head; (g) pacemaker; (h) wound on the area of electrodes’ application; (i) drug consumption; (j) recent application of tDCS; and (k) psychiatric disease impeding understanding of the study. All data were collected at the La Salle University Centre for Advanced Studies. Informed written consent was obtained from all the participants prior to inclusion. All the participants were informed of the study procedures, which were planned under the ethical standards of the Helsinki Declaration.

### Blinding

The participants were blinded to their group allocation. Due to the quasi-experimental design of this study, the assessors were not blinded.

### Interventions

#### Active-tDCS

The active tDCS group received 20 min of tDCS application of 2 mA, given no adverse effects had been reported with a 2 mA intensity application^3,21^. Previous studies have also obtained better results when the tDCS was applied at a 2 mA intensity, increasing the motor evoked potentials amplitude by 40%; this gain had not been reported with a 1 mA stimulation. That outcome suggests a greater motor response of the muscles and axons of the motor descending tracts with a current intensity of 2 mA. The current was gradually increased for 15 s at the beginning and gradually reduced for 15 s at the end.

#### Sham-tDCS

The control group only received the initial 15 s, during which the current intensity increased and then no current flowed during the rest of the application^22^. By using this technique and the fact that having had a tDCS application in the past was an exclusion criterion, we ensured that the participant did not know whether they were receiving either the active or sham intervention.

### Procedures

After giving their consent to partake in the study, all the participants received a set of questionnaires prior to the intervention. These included a sociodemographic assessment as well as an evaluation of their physical activity.

#### Evaluators

Each of the two physiotherapists who were in charge of the measurements received a specific 10-h training session on how to use the instruments. Training was performed with some individuals whose measurements were not included in the study.

#### tDCS intervention

The tDCS technique requires two electrodes. The electrodes used in the tDCS intervention of both groups were symmetrical, with a surface of 50 mm per 70 mm. The anode is the active electrode, and the cathode the referent electrode; they were applied in the following way: anode was placed over M1 and the cathode, was placed over the supra-orbital region. Anodal stimulation requires the anode to be placed where it is intended to facilitate the depolarization of the cells. Through this mode of electrical stimulation, the excitability of the motor region is increased, facilitating neuronal activation^23^.

The Sooma tDCS (Sooma, Helsinki, Finland), was the model we used, given it proposes a placebo protocol, as described previously. The electrodes were saturated with a saline solution to improve current conductivity. Both groups received the exact same measurements, before and after the tDCS application. All variables were measured before and after tDCS application. After the application of tDCS, the measurements were evaluated immediately upon completion.

### Outcome measures

#### Primary outcomes

##### Discriminative sensation

*Tactile acuity threshold* Exacta Von Frey monofilaments (North Coast Medical, Morgan Hill, CA, USA) were used, applying a pressure on the skin until the monofilament bends^24^. The researcher started the assessment with the thinnest monofilament, then gradually increased the diameter until the participant stated that they felt the tactile stimuli. This test was performed three times for each diameter at the thenar eminence of the hand and the back of the foot of the dominant limb. In the hand, the cutaneous pressure threshold has shown an intraclass correlation coefficient (ICC) ranging from 0.96 to 0.99^25^.

#### 2-PD test

The Aesthesiometer Baseline device (AliMed, Deham, MA, USA) was used for the 2-PD test in the same areas of tactile acuity above^26^. Dellon et al. and Mielke et al. had found that 2-PD measurements could be determined with acceptable interobserver reliability and reproducibility in the hand^26,27^.

The participant lay in a supine position. The dominant upper limb remained in external rotation, while the lower limb stayed in a neutral position. The participant’s eyes were closed to ensure they did not know when the examiner brought the monofilaments or the aesthesiometer to his skin. The dominant upper and lower limb were tested.

#### PPT

The PPT was assessed with a Wagner FDX algometer (Wagner Instruments, Greenwich, CT, USA)^28^. The PPT has been defined as the minimal amount of pressure at which a sense of pressure first changes to pain or discomfort. The Wagner FDX algometer consisted of a round rubber disk (area: 1 cm^2^) attached to a pressure (force) gauge. The gauge displayed values in kilograms but given the surface of the rubber tip was 1 cm^2^, the readings were expressed in kg/cm^2^. The pressure was applied at a rate of 0.31 kg/s^29^. Previous studies have reported an intraexaminer reliability of this procedure ranging from 0.6–0.97, whereas the interexaminer reliability ranged from 0.4 to 0.98^30^.

PPTs were tested in two different locations. These sites included the biceps brachii and the rectus femoris. All the assessments were performed in a quiet room. In order to familiarise the participants with the test procedure, pressure was first applied to an area that would not be tested during the study (nondominant forearm). A total of 3 consecutive measurements of the PPT at the 4 locations at intervals of 30 s, and the mean of these 3 trials, were used for the data analysis^30^.

#### Muscle recruitment through electromyographic activity

The activity of surface electromyography (sEMG) during a maximum voluntary isometric contraction was measured^31^. The long head of the biceps brachii was tested, to determine the impact on the upper limb. The rectus femoris was also chosen to assess the effect on the lower limb. Both tests were performed on the participant’s dominant limb.

Both isometric contractions (upper and lower limb) were performed after a clear explanation by the physical therapist. The participant was informed of the procedure, the position to adopt during the contraction, and the exact way to contract to avoid compensating with other muscles and to optimise biceps brachii and rectus femoris fibre recruitment.

The muscle activity during a maximal voluntary contraction was contrasted with inactivity during the resting phase. The participants performed 3 isometric contractions for 5 s, with an equal resting time after each contraction. Throughout this protocol, we were able to record the amplitude changes in sEMG.

**Figure 2:**
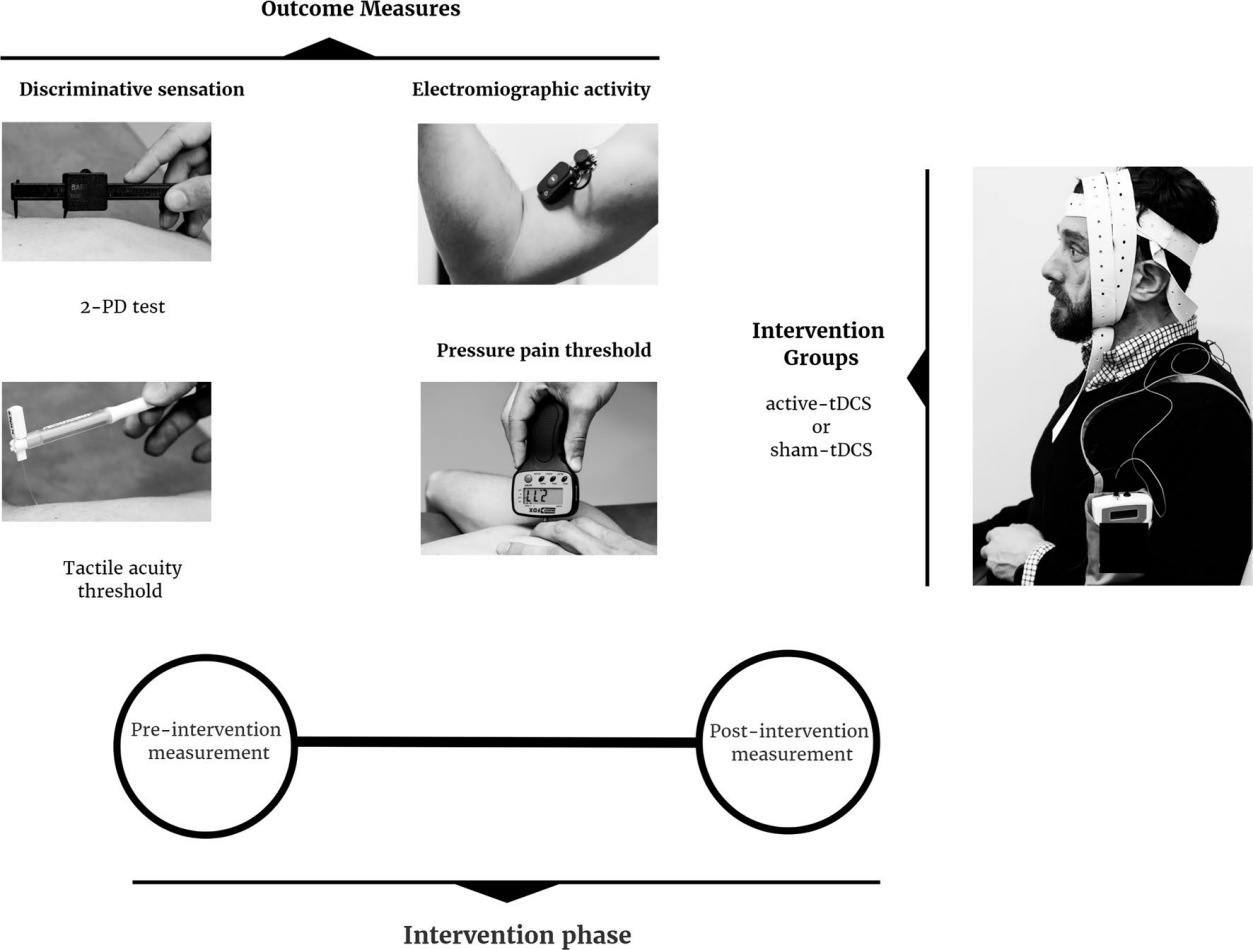
Representation of intervention and the assessments.

The assessment of the lower limb followed the same protocol in the rectus femoris. The electrodes were placed while in a supine position, and the procedure was the same. A Free-EMG 1000 surface electromyography (EMG) device (BTS Bioengineering, Quincy, MA, USA) was used for muscle contraction capture. The signals were filtered using a band-pass filter, and a rectification was performed for signal normalisation. In order to quantify the maximum voluntary contraction, the root mean square (RMS) was calculated, using a fixed window of 0.02 s. In previous studies, the RMS of the EMG had an excellent reliability (ICC = 0.86–0.93) in the lower extremity muscles during bridging and quadruped exercises^32^. The reliability of scapular and arm muscles’ sEMG amplitude values, including the biceps brachii, have been found to be excellent (ICC ≥ 0.75)^33^ (Fig. 2).

#### Baseline outcome

##### Level of physical activity

The level of physical activity was evaluated using the international physical activity questionnaire (IPAQ), which divides the participants into 3 groups according to their level of activity: high, moderate or low/inactive^34^. This questionnaire has shown acceptable validity for measuring total physical activity; its reliability is reported to be approximately 0.65 (r = 0.76; 95% CI 0.73–0.77)^35^.

### Data analysis

The data analysis was performed using the Statistical Package for the Social Sciences (SPSS 25.00, IBM, Chicago, IL, USA). For the data analysis, we used a confidence interval of 95%, considering all those variables with a *p* value of less than 0.05 to be statistically significant. Descriptive statistics used to summarise data for continuous variables are presented as mean ± standard deviation with a 95% confidence interval, and the categorical variables are presented as an absolute number or relative frequency percentage. A repeated measures analysis of variance (ANOVA) was conducted to study the effect on the dependent variables of the between-participant factor ‘intervention group’ with 2 levels (a-tDCS and s-tDCS) and the within-participant factor called ‘time’ with 2 levels (pre- and postintervention). Partial eta squared (ƞ_p_^2^) was calculated as a measure of effect size (strength of association) for each main effect and interaction in the ANOVAs, with 0.01–0.059 representing a small effect, 0.06–0.139 a medium effect and > 0.14 a large effect^36^. The effect size (Cohen’s *d*) was calculated for the main variables. According to Cohen’s method, the effect was considered as small (0.20–0.49), medium (0.50–0.79) or large (> 0.80)^37^.

## Results

A total of 100 healthy participants were included and were allocated into 2 groups of 50 participants per group. No adverse events were reported in either group. No statistically significant preintervention between-group differences in demographic data were found (Table 1).

**Table 1:** Participants’ general characteristics.

Measures	a-tDCS (n = 50)	s-tDCS (n = 50)	p-value
Sex (male/female)	25/29	28/26	0.57
Age (years)	22 ± 2	23 ± 3	0.74
Physical activity			0.16
Sedentary	7	2	
Active	43	48	

Values are shown as the mean ± SD. a-tDCS: experimental group; s-tDCS: control group.

### Discriminative sensation

**Figure 3:**
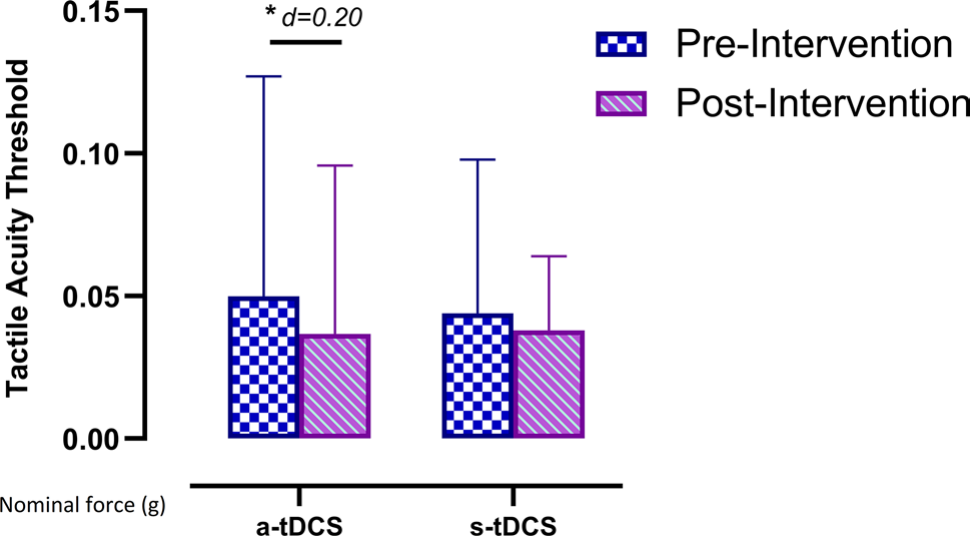
Within-group differences in tactile acuity threshold over thenar eminence of the hand. *a-tDCS *active transcranial direct current stimulation,* s-tDCS *sham transcranial direct current stimulation.

Regarding the tactile acuity threshold assessment in the upper limb, the ANOVA revealed significant changes over time (*F* = 6.28, *p* = 0.014, ƞ_p_^2^ = 0.06) but not for the group*time interaction (*F* = 1.272, *p* = 0.262, ƞ_p_^2^ = 0.013). The post hoc analysis revealed no significant postintervention between-group differences (*p* = 0.832). However, significant within-group pre- and postintervention differences were found in the a-tDCS group, with a small effect size (*p* = 0.012, *d* = 0.20). There were no significant within-group pre- and postintervention differences in the s-tDCS group (*p* = 0.332) (Fig. 3).

Regarding the tactile acuity threshold assessment in the lower limb, the ANOVA revealed no significant differences in group*time (*F* = 3.33, *p* = 0.071, ƞ_p_^2^ = 0.033) nor over time (*F* = 0.229, *p* = 0.633, ƞ_p_^2^ = 0.002).

Regarding the 2-PD assessment in the upper limb, the ANOVA revealed no significant differences in group*time (*F* = 1.037, *p* = 0.311, ƞ_p_^2^ = 0.01) nor over time (*F* = 0.225, *p* = 0.637, ƞ_p_^2^ = 0.002).

With regard to the 2-PD assessment in the lower limb, the ANOVA revealed no significant differences in group*time (*F* = 1.978, *p* = 0.163, ƞ_p_^2^ = 0.02) nor over time (*F* = 2.78, *p* = 0.098, ƞ_p_^2^ = 0.028).

### Pressure pain threshold

Regarding the PPT assessment in the upper limb, the ANOVA revealed no significant differences in group*time (*F* = 0.014, *p* = 0.906, ƞ_p_^2^ = 0.0) nor over time (*F* = 0.977, *p* = 0.325, ƞ_p_^2^ = 0.01).

**Figure 4:**
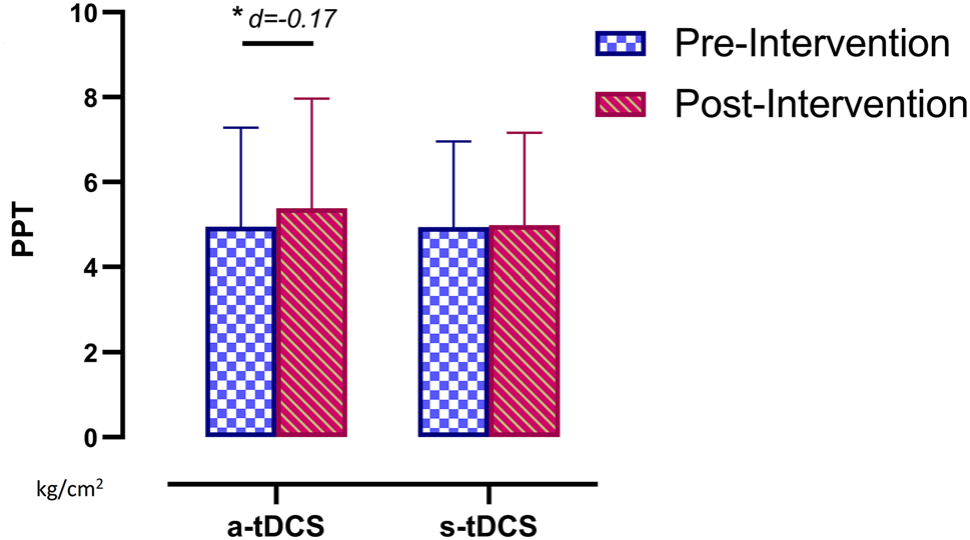
Within-group differences in pressure pain threshold over the rectus femoris. *PPT* pressure pain threshold,* a-tDCS *active transcranial direct current stimulation*; s-tDCS *sham transcranial direct current stimulation.

However, with regard to the PPT assessment in the lower limb, the ANOVA revealed significant differences in group*time (*F* = 4.51, *p* = 0.036, ƞ_p_^2^ = 0.044) and over time (*F* = 6.82, *p* = 0.01, ƞ_p_^2^ = 0.065). The post hoc analysis revealed no significant postintervention between-group differences (*p* = 0.413). However, significant within-group pre- and postintervention differences were found in the a-tDCS group, with a small effect size (*p* = 0.001, *d* =  − 0.17). There were no significant within-group pre- or postintervention differences in the s-tDCS group (*p* = 0.732) (Fig. 4).

### EMG activity

**Figure 5:**
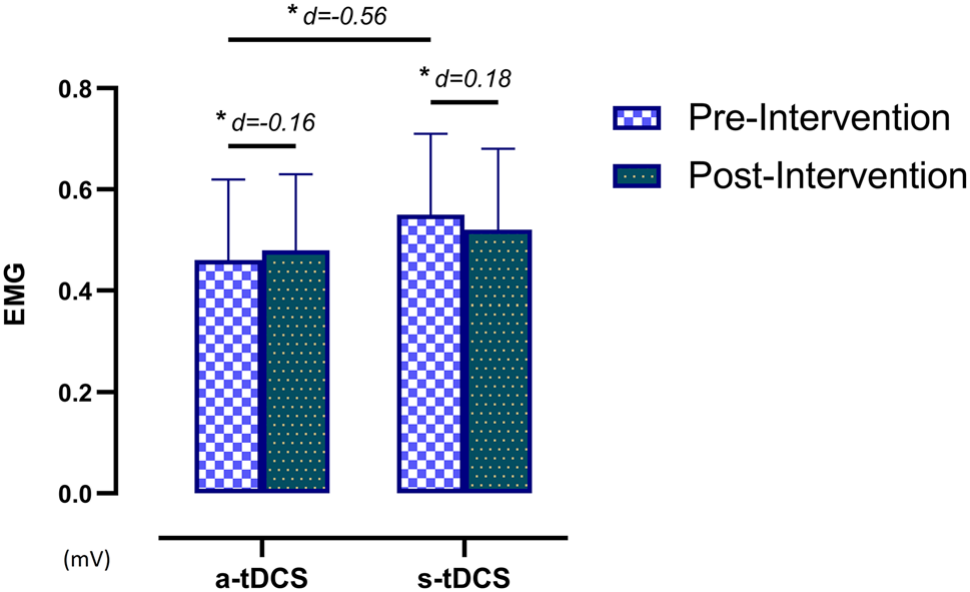
Within- and between-group differences in EMG over the biceps brachii. *EMG* electromyography,* a-tDCS *active transcranial direct current stimulation*, s-tDCS *sham transcranial direct current stimulation.

Regarding the sEMG assessment in the long head of the biceps brachii, the ANOVA revealed significant differences in group*time (*F* = 15.30, *p* < 0.001, ƞ_p_^2^ = 0.135) but not over time (*F* = 0.413, *p* = 0.522, ƞ_p_^2^ = 0.004). The post hoc analysis revealed significant preintervention between-group differences, with a moderate effect size (*p* = 0.004, *d* =  − 0.56) but no significant postintervention between-group differences (*p* = 0.259). In addition, significant pre- and postintervention within-group differences were found both in the a-tDCS group (*p* = 0.023, *d* =  − 0.16) and in the s-tDCS group (*p* = 0.002, *d* = 0.18), with a small effect size (Fig. 5).

**Figure 6:**
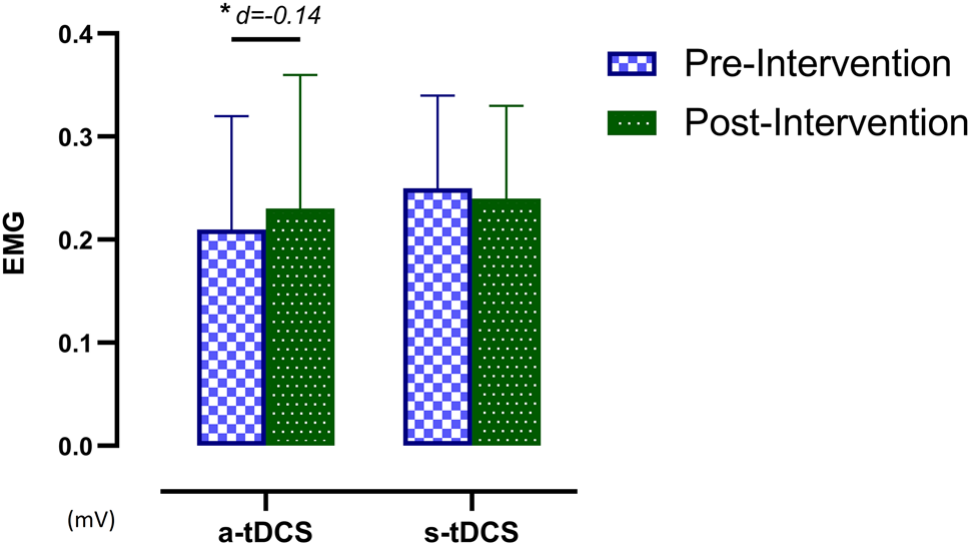
Within-group differences in EMG over the rectus femoris. *EMG* electromyography, *a-tDCS *active transcranial direct current stimulation*, s-tDCS *sham transcranial direct current stimulation.

Finally, regarding the sEMG assessment in the rectus femoris, the ANOVA revealed significant differences in group*time (*F* = 7.12, *p* = 0.009, ƞ_p_^2^ = 0.068) but not over time (*F* = 0.99, *p* = 0.322, ƞ_p_^2^ = 0.01). The post hoc analysis revealed no significant postintervention between-group differences (*p* = 0.795). However, significant pre- and postintervention within-group differences were found in the a-tDCS group, with a small effect size (*p* = 0.011, *d* =  − 0.14). There were no significant pre- and postintervention within-group differences in the s-tDCS group (*p* = 0.239) (Fig. 6).

## Discussion

The main aim of the present study was to assess the short-term effects of a-tDCS in the M1 on sensorimotor variables including discriminative sensation, PPT and EMG activity compared with a sham-tDCS in healthy individuals. Although the results showed some significant within-group differences in the a-tDCS group between the pre- and postintervention interval, none of the results showed that the a-tDCS group had significant between-group differences compared with the sham group.

Regarding EMG activity, anodal stimulation usually increases the excitability of neurons in the area underneath the tDCS scalp electrodes^38^. In our study, a significant increase in the number of muscle fibres recruited during maximal isometric contractions was expected but was not observed, which coincides with Flood et al.^39^ However, a trend towards greater activity was observed in participants with a-tDCS compared with the sham group, which showed a trend towards decreased EMG activity. In fact, although no differences were found between the groups, the within-group differences were statistically significant in the upper limb. Krishnan et al.^40^ showed that anodal tDCS increased the magnitude of biceps brachii activation at 37.5% and 50% of maximum and also resulted in an increase in the peak force and EMG values during maximal muscle contractions in healthy subjects. However, Flood et al.^39^ had found that tDCS did not significantly raise maximal strength production in healthy volunteers. Suzuki et al.^41^ had reported an increase in motor evoked potentials in healthy participants; however, this increase was higher in stroke patients, suggesting that the potential effects of tDCS were different in a clinical population compared with healthy individuals.

Cycling exercises have been studied with the application of tDCS in other brain regions, such as the dorsolateral prefrontal cortex. Holgado et al.^42^ had found that tDCS over the left dorsolateral prefrontal cortex did not affect self-paced exercise performance in trained asymptomatic cyclists. However, Angius et al.^43^ had observed a reduced perception of effort and increased endurance in asymptomatic cyclists following anodal stimulation of the M1 when the cathode was placed on the contralateral shoulder, but not when placed over the prefrontal region. In addition, Vargas et al.^14^ had shown an increase in quadriceps strength during a maximal voluntary contraction after tDCS, yet Giboin et al.^15^ had found the opposite effect. In addition, Hendy et al.^44^ had found that the use of a-tDCS in combination with strength training of the wrist extensor muscles in healthy individuals did not result in improvements to the training itself when combined with a sham tDCS intervention. However, strength training with a-tDCS appears to differentially modulate cortical plasticity, which did not occur following strength training in isolation.

These data suggest that the application of tDCS produces immediate changes in healthy participants although these effects seem small and the data may also suggest that by itself, tDCS is not powerful enough to lead to changes in key variables such as strength or electromyographic activity. However, it appears that it can cause changes in regional neuronal activity during use^45^. Given the findings are somewhat contradictory and unclear, more research is needed.

Regarding the PPT, Borckardt et al.^46^ had reported similar findings as those observed in the present study where no significant effects were found in mechanical PPTs. However, Reidler et al.^47^ had shown that a-tDCS in the M1 significantly increased the PPT in healthy participants. In chronic pain and also in neuropathic pain due to spinal cord injury, the a-tDCS has showed good results in the increase of PPT in combination with other techniques such as TENS and visual illusion respectively^48,49^. In addition, the systematic review and meta-analysis conducted by Vaseghi et al.^50^ found that anodal tDCS stimulation was effective in the increase of PPT in healthy population and also, it showed a decrease of pain level in patients with chronic pain.

Pinto et al.^51^ had reviewed several tDCS studies on chronic pain, and concluded that despite evidence in favour of tDCS in combination with other treatments for chronic pain, it is still not possible to conclude whether tDCS is associated with a useful clinical effect for the treatment of maintained pain. Again, it appears that the results obtained in the present study are controversial, as are those found in the current scientific literature.

Regarding the tactile acuity threshold, the results showed statistically significant within-group differences in the a-tDCS group only in the upper limb. However, these differences were not superior to the s-tDCS group. There is a lack of literature regarding the influence of tDCS on somatosensory variables, especially when the stimulation is in the primary motor cortex. Falcone et al.^52^ had found that the application of tDCS in the prefrontal cortex led to significant improvements in perceptual sensitivity using signal detection theory by distinguishing between the perceptual sensitivity itself and the response bias. These improvements were retained for at least 24 h. Hilgenstock et al.^53^ had studied the effects of tDCS on somatosensory performance and concluded that, compared with a placebo application, tDCS promoted tactile learning by reducing the 2-PD threshold. The main difference with respect to the present study is that they carried out the training over 5 days and not in a single session. Thus, a single session of tDCS might be insufficient to bring about changes in an asymptomatic population.

### Limitations

This study presents several limitations. First, there was no randomisation of the participants, for technical reasons. Second, there was only one session of t-DCS, and in an asymptomatic population. Perhaps with a greater number of sessions different results would have been obtained. Third, due to the design of this study, the assessors were not blinded, and this also should consider as a limitation.

In addition, because tDCS appears to be more powerful in combination with other intervention tools, future studies should be directed towards increasing the number of sessions and combining it with other interventions. A questionnaire to evaluate the effectivity of the sham protocol was not applied and this should be considered as a limitation. Finally, it should be stressed that, although we place the electrode over the M1 as precisely as possible, tDCS is usually placed in a general way over the cerebral cortex.

## Conclusions

Although the results of the present study showed several significant within-group differences in the a-tDCS group, none of the results showed that the a-tDCS group had significant between-group differences compared with the s-tDCS group. However, a single session of anodal stimulation of tDCS in isolation over M1 appears to produce slight changes in the excitability of the primary motor cortex region in healthy individuals although these effects seem very small. These results should be taken with caution due to the small effect sizes obtained.

## Abbreviations

tDCS: Transcranial direct current stimulation
2-PD: Two-point discrimination test
PPT: Pressure pain threshold
EMG: Electromyography
sEMG: Surface electromyography
ANOVA: Analysis of variance
a-tDCS: Active transcranial direct current stimulation
s-tDCS: Sham transcranial direct current stimulation
mA: Milliamperes
